# Delving into the Heterogeneity of Different Breast Cancer Subtypes and the Prognostic Models Utilizing scRNA-Seq and Bulk RNA-Seq

**DOI:** 10.3390/ijms23179936

**Published:** 2022-09-01

**Authors:** Jieyun Xu, Shijie Qin, Yunmeng Yi, Hanyu Gao, Xiaoqi Liu, Fei Ma, Miao Guan

**Affiliations:** Jiangsu Key Laboratory for Biodiversity and Biotechnology, College of Life Sciences, Nanjing Normal University, Wenyuan Road 1, Nanjing 210023, China

**Keywords:** breast cancer, cancer cells, intercellular communication, scRNA-seq, bulk RNA-seq, prognosis

## Abstract

Background: Breast cancer (BC) is the most common malignancy in women with high heterogeneity. The heterogeneity of cancer cells from different BC subtypes has not been thoroughly characterized and there is still no valid biomarker for predicting the prognosis of BC patients in clinical practice. Methods: Cancer cells were identified by calculating single cell copy number variation using the inferCNV algorithm. SCENIC was utilized to infer gene regulatory networks. CellPhoneDB software was used to analyze the intercellular communications in different cell types. Survival analysis, univariate Cox, least absolute shrinkage and selection operator (LASSO) regression and multivariate Cox analysis were used to construct subtype specific prognostic models. Results: Triple-negative breast cancer (TNBC) has a higher proportion of cancer cells than subtypes of HER2+ BC and luminal BC, and the specifically upregulated genes of the TNBC subtype are associated with antioxidant and chemical stress resistance. Key transcription factors (TFs) of tumor cells for three subtypes varied, and most of the TF-target genes are specifically upregulated in corresponding BC subtypes. The intercellular communications mediated by different receptor–ligand pairs lead to an inflammatory response with different degrees in the three BC subtypes. We establish a prognostic model containing 10 genes (risk genes: *ATP6AP1*, *RNF139*, *BASP1*, *ESR1* and *TSKU*; protective genes: *RPL31*, *PAK1*, *STARD10*, *TFPI2* and *SIAH2*) for luminal BC, seven genes (risk genes: *ACTR6* and *C2orf76;* protective genes: *DIO2*, *DCXR*, *NDUFA8*, *SULT1A2* and *AQP3*) for HER2+ BC, and seven genes (risk genes: *HPGD*, *CDC42* and *PGK1*; protective genes: *SMYD3*, *LMO4*, *FABP7* and *PRKRA*) for TNBC. Three prognostic models can distinguish high-risk patients from low-risk patients and accurately predict patient prognosis. Conclusions: Comparative analysis of the three BC subtypes based on cancer cell heterogeneity in this study will be of great clinical significance for the diagnosis, prognosis and targeted therapy for BC patients.

## 1. Introduction

Breast cancer (BC) is the most common malignancy in women, with 2.3 million new cases worldwide in 2020, accounting for one quarter of all cancer cases and one sixth of cancer deaths [1]. BC is a highly heterogeneous tumor and heterogeneity usually exists between subtypes [2]. Clinical classification of BC based on the expression of the estrogen receptor (ER), progesterone receptor (PR) and human epidermal growth factor receptor 2 (HER2) has resulted in three broad subtypes, which are luminal BC, HER2-positive (HER2+) BC and triple-negative breast cancer (TNBC). Among all BC subtypes, luminal BC accounts for approximately 70% [3], TNBC accounts for 15–20% [4] and HER2+ BC accounts for 15–20% [5]. Each BC subtype has unique molecular characteristics, prognosis, clinical behavior, and treatment modalities [6,7,8]. For example, luminal BC patients have a better prognosis and are mainly treated with endocrine therapy and chemotherapy [9]. HER2+ BC has a poor prognosis and rapid progression, and is mainly treated with chemotherapy and anti-HER2 therapy [10]. TNBC is an aggressive subtype with terrible prognosis, strong drug resistance and high mortality. Current therapeutic options are still relatively limited for TNBC [11]. However, the cellular heterogeneity as drivers of cancer progression across different BC subtypes has not been well characterized. Meanwhile, there is still no valid biomarker for predicting the prognosis of BC patients for three subtypes in clinical practice.

Identifying new biomarkers in BC is crucial for accurate prognostic prediction and determining candidate targets of treatment. Delineation of subtypes should be considered when searching for new clinically and prognostically relevant biomarkers due to high heterogeneity across different BC subtypes. Of note, specifically expressed genes in cancer cells of different BC subtypes lead to different clinical phenotypes [12,13,14], which may explain the heterogeneity among different BC subtypes. However, most of the previous studies on BC prognosis-related biomarker mining are based on bulk RNA-seq [15], which only provide the average level of gene expression in different cell populations and do not take into account the widespread transcriptional heterogeneity in different cell populations [16,17]. 

**Figure 1:**
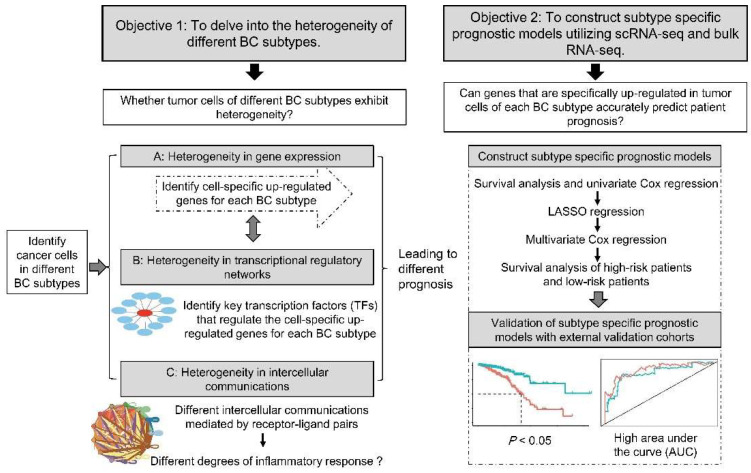
Workflow of delving into the heterogeneity of different breast cancer (BC) subtypes and constructing subtype specific prognostic models. In objective 1, a comparative analysis of three BC subtypes was performed based on the heterogeneity of cancer cells in gene expression, transcriptional regulatory networks and cellular communication. In objective 2, survival analysis, univariate Cox, least absolute shrinkage and selection operator (LASSO) regression and multivariate Cox analysis were used to construct subtype specific prognostic models using cancer cell specific upregulated genes. The accuracy of the prognostic models was then validated using external validation cohorts.

Single-cell RNA sequencing (scRNA-seq) is a promising technology that allows transcriptome analysis of individual cells, identifying different cell types and precisely characterizing the transcription of each cell [18,19,20]. scRNA-seq provides great insight into the diversity of cell states and the heterogeneity of cell populations, making it a useful tool for dissecting the properties of multiple cell types in and around BC tumors. Researchers have used scRNA-seq to analyze the tumor heterogeneity of different subtypes of BC and identified cell clusters associated with poor prognosis or treatment response [21,22]. Single-cell profiles of different immune cells in the BC tumor microenvironment reveal specific immune cell subpopulations that could be potential immunotherapeutic targets [23,24]. In addition, intercellular communication is also one of the central issues in scRNA-seq of BC, as intercellular communication between tumor microenvironments and cells drives cancer progression and influences response to existing therapies [25,26,27].Taken together, the objective of this study was (1) to delve into the heterogeneity of different BC subtypes; and (2) to construct subtype specific prognostic models utilizing scRNA-seq and bulk RNA-seq (Figure 1). In this work, we identified 13,517 cancer cells out of 73,866 cells based on copy number variations (CNVs) in scRNA-seq data from three BC subtype samples. Moreover, we identified specifically upregulated genes and key transcription factors (TFs) in cancer cells of three BC subtypes. Intercellular communications mediated by receptor–ligand pairs between cancer cells and other cells were analyzed. Then, we applied survival analysis, univariate Cox analysis, least absolute shrinkage and selection operator (LASSO) regression analysis and multivariate Cox analysis to construct a prognostic model of each BC subtype using cancer cell specific upregulated genes.

## 2. Materials and Methods

### 2.1. Data Collection and Processing

The scRNA-seq data for this study was downloaded from the Gene Expression Omnibus (GEO) database (GSE176078), and 15 samples including 5 samples from each subtype (luminal BC, HER2+ BC and TNBC) with similar age and high cell numbers were selected (Table S1). Three broad BC subtypes are distinguished by the expression of ER, PR and HER2: luminal BC (ER+, PR+/−), HER2+ BC (HER2+, ER+/−, PR+/−) and TNBC (ER−, PR−).The breast cancer gene expression profiles and corresponding clinical information for the training cohort were obtained from The Cancer Genome Atlas (TCGA) database (https://cancergenome.nih.gov/) on 29 December 2021, containing 678 luminal BC patients, 159 HER2+ BC patients, and 116 TNBC patients after excluding patients with incomplete clinic pathological data. The validation cohort for the subtype of HER2+ BC was derived from the METABRIC dataset (http://www.cbioportal.org/) on 6 March 2022, including 220 HER2+ BC patients. The validation cohort for subtypes of luminal BC and TNBC were downloaded from the GEO database (GSE25066) containing 143 and 122 patients, respectively. All BC patients in the validation cohort have complete survival information.

### 2.2. Single-Cell Data Integration and Analysis

The Seurat package (version 4) was used to integrate scRNA-seq. Cells with the number of expressing genes below 200 or above 8000 were removed. In addition, cells with mitochondrial content greater than 20% were filtered out, and a total of 73,866 cells with single-cell transcriptome data were obtained for further analysis. The Harmony algorithm was used to eliminate batch effects. The top 2000 feature variables with the highest variance were selected for subsequent descending and clustering. Uniform manifold approximation and projection (UMAP) dimensionality reduction was used to project cells in two dimensions. The AddModuleScore function in the Seurat package was used to evaluate the gene set scores of the cells [28,29]. Gene sets in the inflammatory response hallmark were mainly obtained from the msigdbr R package [30]. CellPhoneDB software was used to analyze intercellular communications and discover significant receptor–ligand pairs; only highly interacting receptor–ligand pairs were preserved with *p* value < 0.01 and means > 0.6 [31].

### 2.3. Identification of Cancer Cells by Calculating Cell Copy Number Variations

InferCNV [32] was used to infer large-scale CNVs in single-cell gene expression data. Control cells which contain 500 B cells, 500 T cells and 500 endothelial cells were randomly selected, serving as cells with a normal copy number, and the random seed was set to 123 to ensure reproducibility [33]. Normal epithelial cells and cancer cells were separated by checking the iterative clustering effect and calculating the copy number variation score [34,35].

### 2.4. Identification and Functional Analysis of Differentially Expressed Genes

The FindMakers function in the Seurat package was used to find the differentially expressed genes with filter criteria (|logFC| > 0.25 and FDR < 0.01). In each BC subtype, overlapped genes differentially upregulated in cancer cells compared with the normal epithelial cells group, and differentially upregulated compared with cancer cells of other subtypes group, were defined as cancer cell-specific upregulated genes. The ClusterProfiler [36] package was used for Gene Ontology (GO) biological process (BP) analysis. Significantly enriched GO BP terms were identified by a *q* value smaller than 0.05. 

### 2.5. Inference of Gene Regulatory Networks (GRNs) in Tumor Cells

SCENIC [37] workflow was applied to normalize expression matrices of 5000 randomly selected epithelial cells to infer transcriptional regulatory networks in tumor cells of the three BC subtypes. Firstly, GEne Network Inference with Ensemble of trees (GENIE3) [38] was utilized to infer the co-expression modules between TFs and candidate target genes. Secondly, RcisTarget was used to identify TFs and the genes that they directly regulate. Finally, each formed regulon was scored in each cell using AUCell [39]. Key TFs for tumor cells of each BC subtype were identified with *p* < 0.01. The transcriptional regulatory networks of key TFs were visualized via Cytoscape software.

### 2.6. Construction of Prognostic Risk Model

Patients in the TCGA database with a follow-up time of more than 30 days were included to establish a prognostic risk model. To screen for genes associated with prognosis in BC subtypes, batch screening was performed by survival analysis and univariate Cox regression, and *p* values less than 0.05 were included. To screen for the most significant genes affecting overall survival (OS) in the three BC subtypes, LASSO regression analysis was used to remove genes with collinear correlations to reduce the number of prognostic genes. Finally, the remaining genes were subjected to multivariate Cox regression to screen independent prognostic genes and construct the prognostic model. The mathematical formula for calculating risk score is: Risk score = h_0_(t) × exp (β_1_x_1_ + β_2_x_2_ +… + β_n_x_n_). Herein, h_0_(t) is a constant, x_n_ represents the prognostic genes, exp represents the expression level of these genes, β_n_ represents the multiple regression coefficients of the prognostic genes. Survival and survminer packages were used to construct survival curves to evaluate survival differences between high-risk and low-risk groups. The discriminant ability of the model was evaluated by the receiver operating characteristic (ROC) curve [40].

### 2.7. Data Statistics and Visualization

All graph constructions in this study were performed using R package software (version 4). The Wilcox test was used to determine statistical differences in different groups. Survival curves were measured using the Kaplan–Meier method, and the statistical significance of differences was determined using the log-rank test. ROC curves were used to assess the predictive power of the prediction models. *p* < 0.05 was defined as statistically significant.

## 3. Results

### 3.1. TNBC Has a Higher Proportion of Tumor Cells

**Figure 2:**
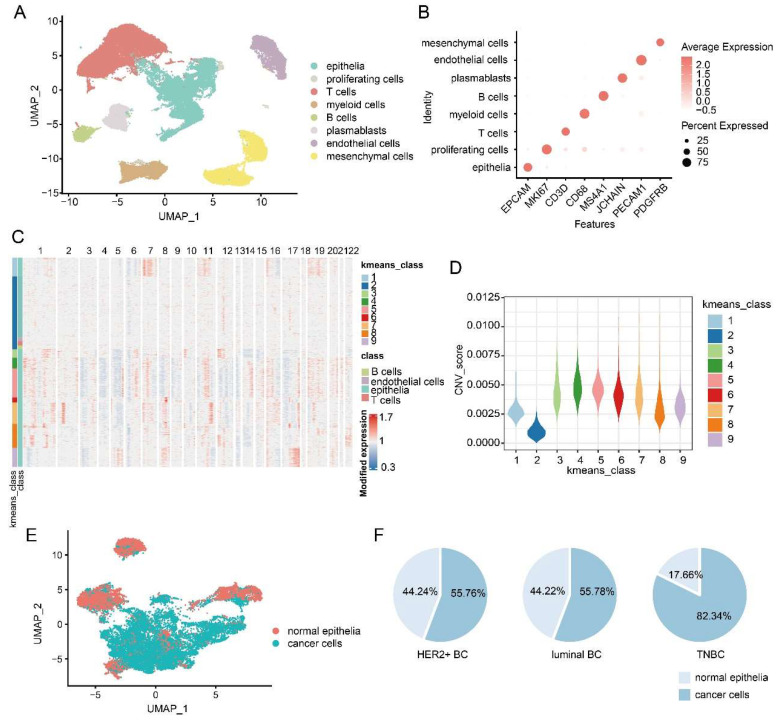
Identification of tumor cells from epithelia. (**A**) Uniform manifold approximation and projection (UMAP) cluster plot of different BC cell types. (**B**) Expression levels of cellular markers corresponding to different BC cell types. (**C**) Unsupervised clustering of inferred large-scale copy number variations (CNVs) to identify cancer cells from epithelia cells. Epithelial cells and reference cells (B cells, T cells and endothelial cells) are in the y-axis and chromosomal regions in the x-axis. (**D**) Violin plot showing the differences of CNVs scores among 9 clusters. (**E**) UMAP cluster plot showing the distribution of normal epithelial cells and cancer cells. (**F**) The proportion of cancer cells and normal epithelial cells of the three subtypes of BC.

After integrating single-cell data from different BC subtypes, 73,866 cells from 15 samples were obtained, and eight major cell types including epithelial cells (*EPCAM*+), proliferating cells (*MKI67*+), T cells (*CD3D*+), myeloid cells (*CD68*+), B cells (*MS4A1*+), plasma cells (*JCHAIN*+), endothelial cells (*PECAM1*+) and mesenchymal cells (*PDGFRB*+) were identified by cell markers (Figure 2A,B). All markers and cell types have good specificity, indicating that the identification of cell types is accurate and efficient (Figure 2B). Cancer cells of BC originate from epithelial cells, so we defined cancer cells in all epithelial cells by calculating CNVs inferred from single-cell gene expression data with reference cells of immune cells (B cells and T cells) and endothelial cells. Unsupervised clustering with nine clusters was applied to distinguish cells with high CNVs and low CNVs (Figure 2C). As expected, CNVs levels in most epithelial clusters were obviously higher than reference cells. The second cluster containing reference cells (immune cells and endothelial cells) have the lowest coefficient of variation among the nine clusters (Figure 2D). Therefore, the epithelial cells in the second cluster were defined as normal epithelial cells, and the epithelial cells in other clusters were defined as cancer cells. A total of 13,517 cancer cells out of 19,534 epithelial cells were identified, indicating that cancer cells accounted for a large proportion (69.2%) of BC epithelial cells (Figure 2E). Among the three subtypes of BC, TNBC had the highest proportion of cancer cells in epithelial cells, up to 82.34%, while luminal BC (55.78%) and HER2+ BC (55.76%) had a similar relative lower proportion than TNBC (Figure 2F). 

### 3.2. Functions of Specifically Upregulated Genes and Variation of GRNs in Three BC Subtypes

**Figure 3:**
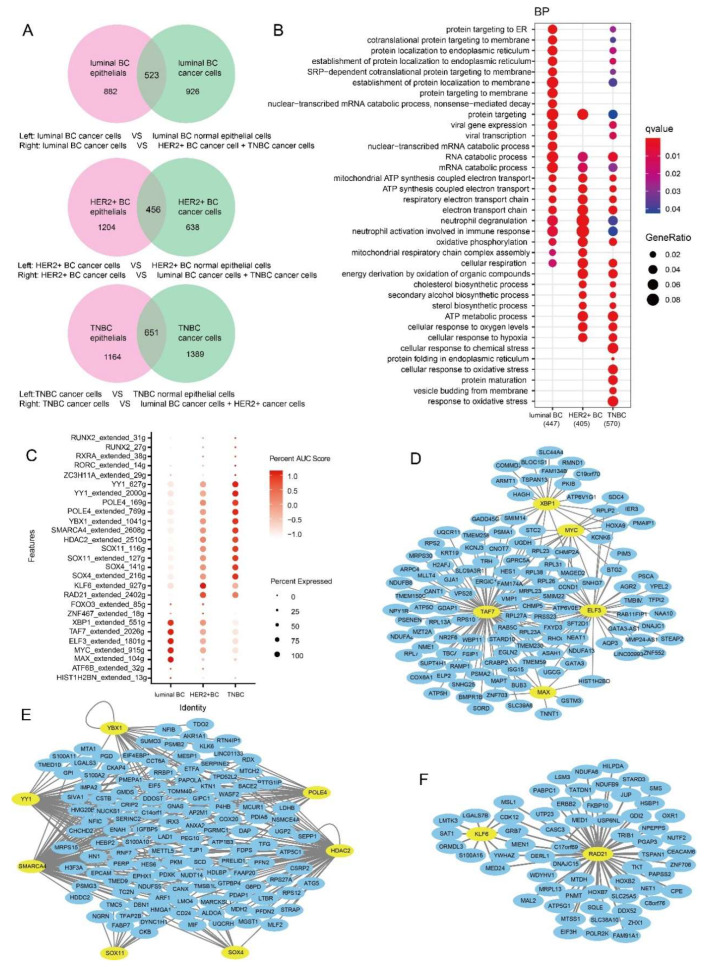
Functions and gene regulatory networks of subtype-specific upregulated genes. (**A**) Intersection of differentially upregulated genes in cancer cells compared with normal epithelial cells (**left**) and differentially upregulated compared with other subtypes (**right**). (**B**) GO biological process function enrichment of specific upregulation genes in cancer cells of three BC subtypes. (**C**) Dotplot showing TFs specifically enriched in cancer cells of different BC subtypes. (**D**–**F**) Key TFs and their highly upregulated target genes in (**D**) luminal BC, (**E**) TNBC and (**F**) HER2+ BC tumor cells.

The number of genes specifically upregulated in cancer cells was 523, 456 and 651 for subtypes of luminal BC, HER2+ BC and TNBC, respectively (Figure 3A). GO enrichment analysis demonstrated that the specific upregulated genes in the three BC subtypes were related to functions such as protein targeting, RNA catabolic processes, cellular respiration, mitochondrial ATP synthesis coupled electron transport, and the respiratory electron transport chain, all of which are essential for the survival of cancer cells (Figure 3B). In addition, these specific upregulated genes in three BC subtypes were also associated with neutrophil degranulation and neutrophil activation (Figure 3B). Neutrophils are the first responders to inflammation and infection, suggesting that there is an inflammatory response in BC. Moreover, pathways of protein targeting to membrane and the nuclear-transcribed mRNA catabolic process were enriched by specific upregulated genes of luminal BC cancer cells compared to HER2+ BC and TNBC. The specific upregulated genes in tumor cells of HER2+ BC and TNBC were associated with energy derivation by the oxidation of organic compounds, ATP metabolic process, cholesterol biosynthetic process, secondary alcohol biosynthetic process, and sterol biosynthetic process (Figure 3B). These pathways modulated by specific upregulated genes of cancer cells demonstrated that cancer cells need more energy to synthesize more substances. Most importantly, we found that cancer cells of HER2+ BC and TNBC, especially TNBC, specifically expressed genes that responded to chemical and oxidative stress compared with luminal BC and HER2+ BC, which were related to the high drug resistance of TNBC (Figure 3B) [41,42,43]. 

We used the SCENIC algorithm to analyze single cell transcriptional regulatory networks in tumor cells to identify the enriched key TFs of the three BC subtypes (Figure 3C). We found that XBP1, TAF7, ELF3, MYC, MAX, etc., were more enriched in tumor cells of luminal BC than the other two subtypes. The highly active TFs in TNBC were YY1, YBX1, SOX11, SOX4, POLE4, SMARCA4 and HDAC2, etc., while in HER2+ BC they were FOXO3, ZNF467, and especially RAD21 and KLF6 (Figure 3C). Since TFs enriched in BC subtypes may regulate gene-specific expression, we further identified overlapping genes between the target genes of TFs and cancer cell-specific upregulated genes of different BC subtypes. There were 317, 151 and 512 upregulated target genes for subtypes of luminal BC, HER2+ BC and TNBC, respectively. Highly upregulated target genes with avg_log2FC > 0.5 were visualized in Figure 3D,F.

### 3.3. Intercellular Communication of Cancer Cells Leads to BC Inflammatory Microenvironment

**Figure 4:**
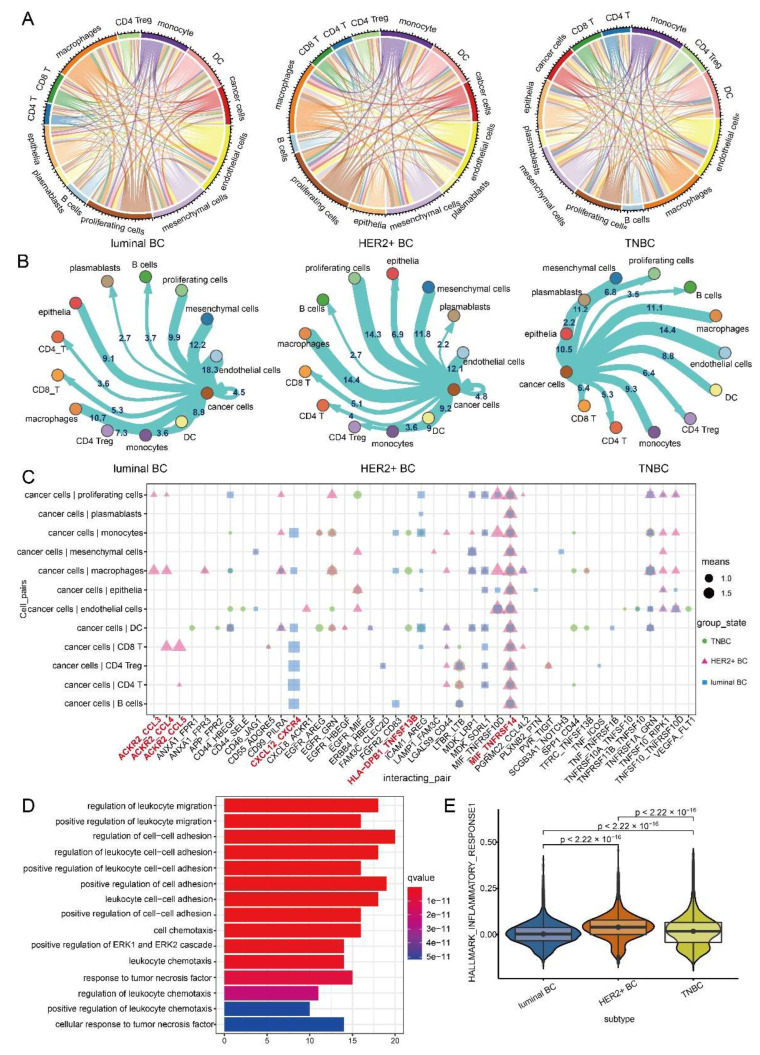
Cell communication of cancer cells leads to BC inflammatory microenvironment. (**A**) Overall intercellular communication profiles of the three BC subtypes. (**B**) Intercellular communication frequency maps of cancer cells of three BC subtypes. (**C**) Receptor–ligand pairs for communication between cancer cells and other cells of the three BC subtypes. (**D**) GO biological process enrichment analysis of receptor–ligand genes for communication between BC cancer cells and other cells. (**E**) Scores of cellular inflammatory responses in the three BC subtypes.

We used the CellphoneDB algorithm to analyze the intercellular communication of three BC subtypes (Figure 4A and Figure S1). The top cell types with the highest frequency of communication with cancer cells were mesenchymal cells and endothelial cells in luminal BC, macrophages and proliferating cells in HER2+ BC, and plasmablasts and endothelial cells in TNBC, respectively (Figure 4B). We also analyzed the ligand–receptor pairs with high interactions between cancer cells and other cells in three BC subtypes (Figure 4C). We found that these receptor and ligand genes were significantly enriched in leukocyte migration, cell chemotaxis, and tumor necrosis factor response (Figure 4D), and also the inflammatory character of the BC tumor microenvironment was consistent with our previous results that there is an inflammatory response in BC. Among the receptor–ligand pairs with high interactions, cancer cells of the three BC subtypes communicate with all other cell types through the MIF-TNFRSF14. Moreover, cancer cells of the luminal BC communicate with immune cells through CXCL12_CXCR4. Cancer cells of HER2+ BC communicate with macrophages and CD8 T cells through receptor–ligand pairs of ACKR2-CCL3, ACKR2-CCL4 and ACKR2-CCL5 (Figure 4C). Cancer cells of TNBC communicate with macrophages and DC through the receptor–ligand pair of HLA−DPB1_TNFSF13B (Figure 4C). Interestingly, most of these ligands and receptors are involved in the induction of inflammatory responses, which suggest that receptors and ligands that communicate between cancer cells and other cells play a key role in inducing inflammatory responses. We also speculate that the different ligand–receptor pairs that communicate between tumor cells and other cells to trigger an inflammatory response will also lead to different degrees of inflammatory response in the three BC subtypes. As expected, among the three BC subtypes, the inflammatory response scores of TNBC and HER2+ BC were significantly higher than those of luminal BC (Figure 4D).

### 3.4. Prognostic Model Construction Using Cancer Cell-Specific Upregulated Genes

**Figure 5:**
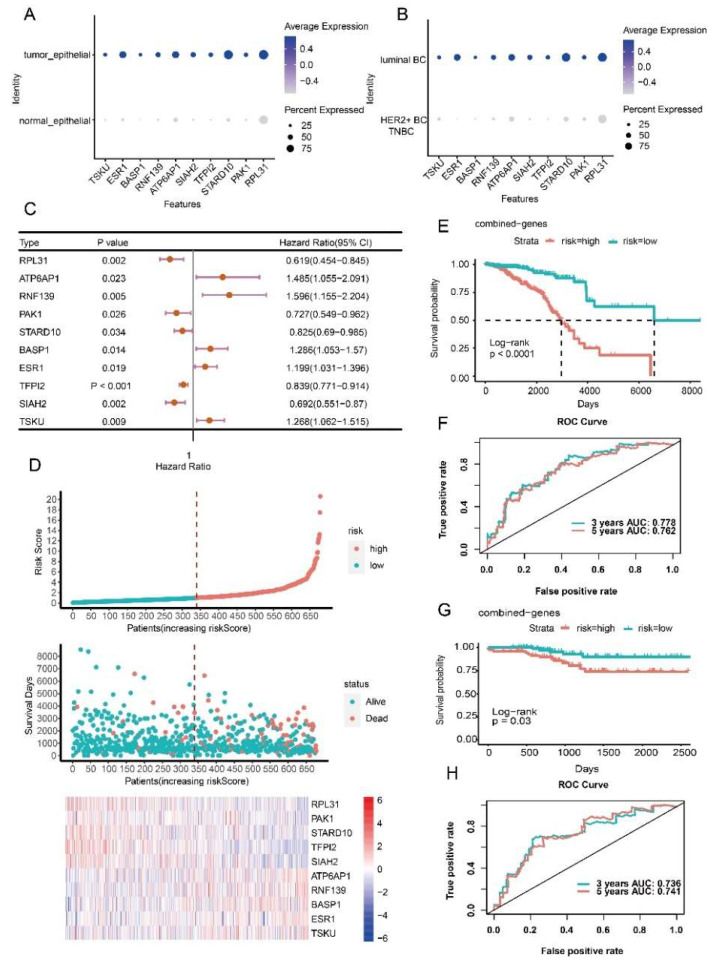
Construction and evaluation of the prognostic risk model for luminal BC. (**A**) Expression levels of prognostic factors between normal and cancer cells. (**B**) Expression levels of prognostic factors in cancer cells of luminal BC and other subtypes. (**C**) HR and *p* values of prognostic factors by univariate Cox regression. (**D**) Risk curves and scatter plots of sample survival probability for each sample reordered by risk score, heatmap of expression of prognostic factors in low-risk and high-risk groups. (**E**) Differences in overall survival between high-risk and low-risk groups in the training cohort of luminal BC. (**F**) Receiver operating characteristic curve (ROC) analysis of the sensitivity and specificity of the prognostic model in the training cohort of luminal BC. (**G**) Differences in overall survival between high-risk and low-risk groups in the external validation cohort of luminal BC. (**H**) ROC analysis of the sensitivity and specificity of the prognostic model in the external validation cohort of luminal BC.

**Figure 6:**
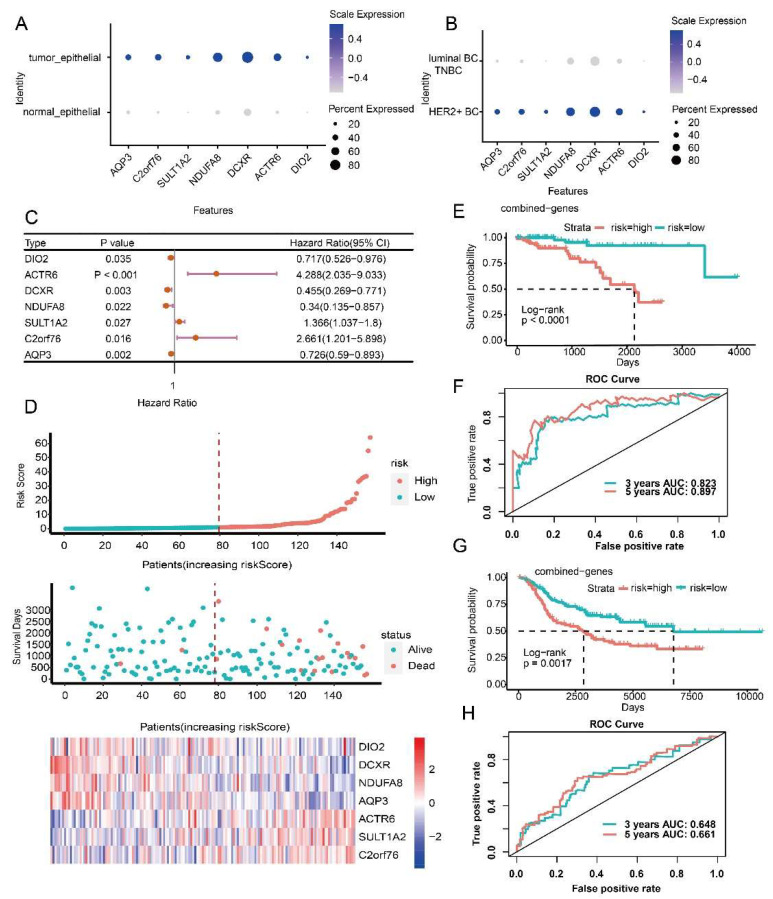
Construction and evaluation of the prognostic risk model for HER2+ BC. (**A**) Expression levels of prognostic factors between normal and cancer cells. (**B**) Expression levels of prognostic factors in cancer cells of HER2+ BC and other subtypes. (**C**) HR and *p* values of prognostic factors by univariate Cox regression. (**D**) Risk curves and scatter plots of sample survival probability for each sample reordered by risk score, heatmap of expression of prognostic factors in low-risk and high-risk groups. (**E**) Differences in overall survival between high-risk and low-risk groups in the training cohort of HER2+ BC. (**F**) Receiver operating characteristic curve (ROC) analysis of the sensitivity and specificity of the prognostic model in the training cohort of HER2+ BC. (**G**) Differences in overall survival between high-risk and low-risk groups in the external validation cohort of HER2+ BC. (**H**) ROC analysis of the sensitivity and specificity of the prognostic model in the external validation cohort of HER2+ BC.

**Figure 7:**
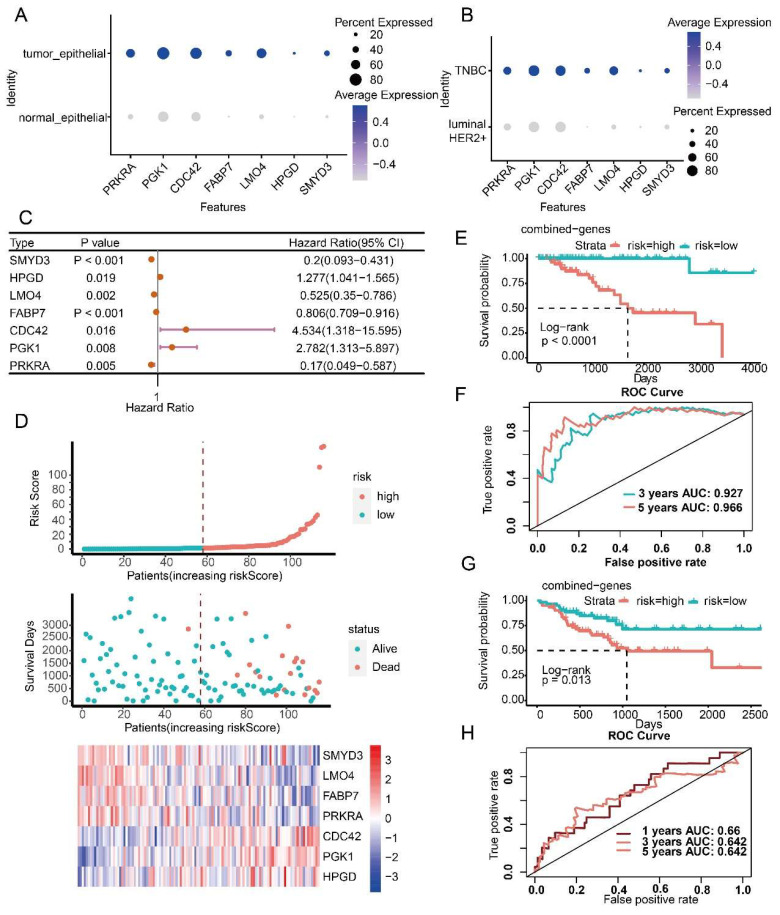
Construction and evaluation of the prognostic risk model for TNBC. (**A**) Expression levels of prognostic factors between normal and cancer cells. (**B**) Expression levels of prognostic factors in cancer cells of TNBC and other subtypes. (**C**) HR and *p* values of prognostic factors by univariate Cox regression. (**D**) Risk curves and scatter plots of sample survival probability for each sample reordered by risk score, heatmap of expression of prognostic factors in low-risk and high-risk groups. (**E**) Differences in overall survival between high-risk and low-risk groups in the training cohort of TNBC. (**F**) Receiver operating characteristic curve (ROC) analysis of the sensitivity and specificity of the prognostic model in the training cohort of TNBC. (**G**) Differences in overall survival between high-risk and low-risk groups in the external validation cohort of TNBC. (**H**) ROC analysis of the sensitivity and specificity of the prognostic model in the external validation cohort of TNBC.

Three prognostic risk models for three BC subtypes (luminal BC, HER2+ BC and TNBC) were obtained using survival analysis, univariate Cox regression, LASSO regression analysis, and multivariate Cox regression. The prognostic risk model of luminal BC included ten genes (*RPL31*, *PAK1*, *STARD10*, *TFPI2*, *SIAH2*, *ATP6AP1*, *RNF139*, *BASP1*, *ESR1* and *TSKU*) (Figure S2). Seven genes (*DIO2*, *ACTR6*, *DCXR*, *NDUFA8*, *SULT1A2*, *C2orf76* and *AQP3*) were contained in the prognostic risk model of HER2+ BC (Figure S3). Seven combinations (*SMYD3*, *HPGD*, *LMO4*, *FABP7*, *CDC42*, *PGK1* and *PRKRA*) constructed the prognostic risk model of TNBC (Figure S4). These prognostic factors were specific differentially upregulated in cancer cells compared with normal epithelial cells and differentially upregulated compared with the cancer cells of the other subtypes (Figure 5A,B, Figure 6A,B and Figure 7A,B). Among genes in the prognostic risk model for luminal BC, *RPL31*, *PAK1*, *STARD10*, *TFPI2* and *SIAH2* were protective factors, while *ATP6AP1*, *RNF139*, *BASP1*, *ESR1* and *TSKU* were risk factors. *DIO2*, *DCXR*, *NDUFA8* and *AQP3* genes were protective factors of HER2+ BC, while *ACTR6*, *SULT1A2* and *C2orf76* genes were risk factors. *SMYD3*, *LMO4*, *FABP7* and *PRKRA* were protective factors of TNBC, while *HPGD*, *CDC42* and *PGK1* were risk factors (Figure 5C, Figure 6C and Figure 7C).

Based on the established prognostic risk model of each BC subtype, patients were divided into high risk and low risk groups according to the median risk score. The results showed that in the three BC subtypes, the mortality rate of the high-risk group was higher than that of the low-risk group, and the expression of risk factors increased with the increase of risk value, while the protective factors decreased with the increase of risk value (Figure 5D, Figure 6D and Figure 7D). As expected, the overall survival of the high-risk group was worse than that of the low-risk group (*p* < 0.0001) (Figure 5E, Figure 6E and Figure 7E). To evaluate the performance of the prognostic risk model, the ROC curve was drawn to confirm the prognostic accuracy of the risk score. The area under the curve (AUC) of the prognostic risk model were 0.778 and 0.762 for luminal BC, 0.823 and 0.897 for HER2+ BC, 0.927 and 0.966 for TNBC at 3-years and 5-years, respectively (Figure 5F, Figure 6F and Figure 7F). These results illustrate the outstanding predictive performance of the three prognostic models of specific upregulated gene composition. We also obtained results in the external validation cohort that were consistent with the training cohort; the overall survival of the high-risk group was significantly worse than that of the low-risk group with *p* smaller than 0.05 (Figure 5G, Figure 6G and Figure 7G). The AUC of the prognostic risk model in the validation cohort were 0.736 and 0.741 for luminal BC, 0.648 and 0.661 for HER2+ BC at 3 and 5 years, and 0.66 and 0.642 for TNBC at 1 and 3 years, respectively (Figure 5H, Figure 6H and Figure 7H). Above all, these results suggest that the three prognostic models are accurate and reliable.

## 4. Discussion

BC is the most common malignant tumor with high heterogeneity in women around the world and the main cause of cancer-related death in women [44,45]. The high heterogeneity of BC presents a challenge for classification and treatment. Exploring the heterogeneity of different BC subtypes is considered a key step towards the goal of subtype-specific targeted therapy in BC clinical practice [46]. In this study, cancer cells were identified from scRNA-seq data of BC samples based on CNVs. We illustrate the heterogeneity of cancer cells in gene expression, transcriptional regulatory networks, and the intercellular communications of different BC subtypes, which lead to different clinical performance and prognosis of corresponding BC subtypes (Figure 1).

We found that TNBC had a higher proportion of cancer cells than other subtypes. Obviously, the high proportion of cancer cells is associated with difficulty in treating and susceptibility to recurrence of TNBC. The number of specifically upregulated genes in cancer cells of luminal BC, HER2+ BC and TNBC were 523, 456 and 651, respectively. Functional analysis of these genes demonstrated that cancer cells of HER2+ BC and TNBC, especially TNBC, expressed genes in antioxidant and anti-chemical stress pathways compared with that of luminal BC. Cancer cells grow in a hypoxic environment, and reactive oxygen and nitrogen species (RONS) are elevated in a variety of cancer cells under hypoxia [41]. The cytotoxicity of anticancer drugs such as tamoxifen, paclitaxel and As_2_O_3_ is associated with the accumulation of O_2_, H_2_O_2_ and NO [42,43]. The chemical resistance of cancer cells to these anticancer drugs is proportional to the activity of antioxidant genes. The unique expression of antioxidant and anti-chemical stress genes in TNBC explains the high drug resistance of TNBC compared with other BC subtypes, which may provide new ideas for the treatment of TNBC.

TFs have long been recognized as important aspects in maintaining cellular identity and function, and the increasing or decreasing expression of TF can significantly affect cellular function [47]. The results of the transcriptional regulatory networks in tumor cells of three BC subtypes indicate that the key TFs in tumor cells of different BC subtypes varied. TFs, such as XBP1, TAF7, ELF3, MYC, MAX, etc., were more enriched in tumor cells of luminal BC than the other two subtypes. The highly activated TFs in TNBC were YY1, YBX1, SOX11, SOX4, POLE4, SMARCA4 and HDAC2, etc., while in HER2+ BC they were FOXO3, ZNF467, and especially RAD21 and KLF6. Most of the target genes regulated by these key TFs are the specific enriched and upregulated genes of corresponding subtypes, revealing that these TFs might be of great potential value in breast cancer treatment and drug development. Our TF enrichment results in tumor cells are consistent with many previous studies which demonstrated that TF of YY1 could drive many aggressive cancer phenotypes [48]. The TF of YBX1 is upregulated in TNBC and plays a role in TNBC invasion by regulating glycolysis and EMT-related gene expression [49]. The TF of SOX11 is a critical regulator of basal-like breast cancer growth, invasion, and basal-like gene expression [50]. The TF of MYC is a cancer driver that regulates many biological activities that contribute to tumorigenesis [51]. SOX4 increases breast cancer cell viability, migration, and invasion in vitro, and enhances tumor growth and metastasis in vivo [50]. SMARCA4 is closely related to tumor immune evasion [52]. HDAC3 is strongly expressed in a subgroup with more aggressive breast cancer [53]. Nuclear expression of XBP1s correlates with shorter breast cancer survival [54]. RAD21 expression is associated with early recurrence and treatment resistance in sporadic breast cancer [55]. 

Genes specifically upregulated in cancer cells of luminal BC, HER2+ BC and TNBC were all enriched in neutrophil degranulation and neutrophil activation pathways. Neutrophil granule protein released after neutrophil activation is associated with tumor progression, and this differential granule mobilization may lead to the migration of neutrophils and associated cancer cells [56]. Meanwhile, neutrophils are the first responders of inflammation and infection [57], and the activation of neutrophils suggests the existence of inflammatory reactions in BC tumor tissues, which was consistent with the results from intercellular communications analysis. 

Receptor–ligand pairs that mediate cellular communication between cancer cells and other cells are significantly enriched in the pathways of leukocyte migration, tumor necrosis factor response, and cell chemotaxis. Cancer cells in all three BC subtypes communicate with all other cell types through the MIF-TNFRSF14. TNFRSF14 is a membrane-bound receptor that activates the NF-κB pathway, leading to a pro-inflammatory response [58,59]. Cancer cells of luminal BC communicate with immune cells through CXCL12_CXCR4. The CXCLs_CXCR family is known to induce neutrophil recruitment in the inflammatory response [60]. Cancer cells of HER2+ BC communicate with macrophages and CD8_T cells through ACKR2-CCL3, ACKR2-CCL4 and ACKR2-CCL5. CCL3, CCL4 and CCL5 are pro-inflammatory chemokines, which are mainly used as attractants of leukocytes (monocytes and neutrophils) and are considered to mediate chronic and acute inflammation [61]. Cancer cells of TNBC communicate with macrophages and DC through the receptor–ligand pair of HLA−DPB1_TNFSF13B. TNFSFs are key molecules in local and systemic inflammatory networks [62]. These results suggest that receptor–ligand pairs for communication between cancer cells and other cells play a key role in inducing inflammatory responses in BC tissues. Inflammation is related to the occurrence and malignant progression of most cancers, and the malignant progression of cancer cells can be promoted through the recruitment and activation of inflammatory cells. Both external and internal inflammation can lead to immunosuppression, thus providing a preferred environment for the development of cancer cells [63,64,65]. The different ligand–receptor pairs communicate between tumor cells and other cells, triggering inflammatory response with different degrees in the three BC subtypes. Among the three BC subtypes, the inflammatory response scores of TNBC and HER2+ BC were significantly higher than those of luminal BC. Obviously, the high inflammatory response of TNBC and HER2 + BC is inseparable from the poor prognosis of TNBC and HER2+ BC. Our study shows that the heterogeneity exhibited by cancer cells of the three BC subtypes is correlated with the clinical prognosis of each subtype, suggesting the contribution of heterogeneity from cancer cells in shaping subtype heterogeneity.

We combined bulk RNA-seq and scRNA-seq to construct subtype specific prognostic models using cancer cell-specific upregulation genes. Three prognostic models can distinguish high-risk patients from low-risk patients and effectively predict the survival rate of patients, although there is no direct association between prognostic factors for each BC subtype (Figure S5). There were some significant enriched GO terms of these prognostic genes for each subtype, but most terms only contain very few genes. (Figure S5). Moreover, prognostic factors of three BC subtypes were able to distinguish the three BC subtypes to some extent, especially luminal BC and TNBC. We suspect the poor differentiation for TNBC is due to the high heterogeneity of TNBC (Figure S6). Based on our subtype-specific prognostic models, *HPGD*, *CDC42,* and *PGK1* are the risk factors of TNBC, *ACTR6*, *SULT1A2* and *C2orf76* are the risk factors of HER2+ BC, while *ATP6AP1*, *RNF139*, *BASP1*, *ESR1* and *TSKU* are the risk factors of luminal BC. *HPGD,* which encodes alcohol dehydrogenase, was reported as a marker of poor prognosis of breast cancer [66]. The overexpression of *CDC42* is associated with poor prognosis in BC because *CDC42* enhances the migration of cancer cells [67]. *PGK1* overexpression is associated with the mutations of common tumor suppressor genes *TP53* and *CDH1* [68]. *ACTR6* can be used as a marker of poor prognosis in lung cancer [69]. Overexpression of *SULT1A2* in BC tissues is significantly correlated with BC staging [70]. High expression of *ATP6AP1* in breast cancer is associated with poor prognosis [71]. *RNF139* is a driver gene closely associated with breast cancer [72]. *BASP1* was reported to be highly expressed in cancer and promotes the proliferation of cancer [73]. *ESR1* mutation has become a key mechanism of endocrine therapy resistance in breast cancer [74]. High expression of *TSKU* is associated with a poor prognosis of NSCLC patients [75]. Above all, these risk factors may serve as targets for BC treatment in addition to prognosis of the survival of BC patients. Our study continues the basic, but broadly applicable, clinical subtype classification of the data source (luminal BC, HER2+ BC and TNBC) [76]. Of note, luminal BC can be subdivided into luminal A, luminal B and luminal-HER2 groups [77], and the accurate classification of them needs to refer to more indicators such as KI67, CCNB1, MKI67 and MYBL2 [78]. It is still difficult for us to simply distinguish ER+ into luminal A and luminal B based on the existing information. Taking luminal BC as a subtype contributes to the search for representative biomarkers which are broadly applicable to luminal BC, but it has limitation in exploring the heterogeneity of breast cancer. We hope that more clinical data will be available to explore the heterogeneity of luminal BC in the future.

## 5. Conclusions

In this study, we selected and reanalyzed scRNA-seq data of cancer cells from three BC subtypes. The proportion of cancer cells in TNBC was higher than that of HER2+ BC and luminal BC. There exists heterogeneity both in GRNs and intercellular communication in cancer cells of different BC subtypes. Cancer cells of TNBC subtype uniquely upregulated genes were related to antioxidant and anti-chemical stress, which are associated with high drug resistance and poor prognosis in TNBC. The intercellular communication of cancer cells leads to the inflammatory response of breast tissue, and the different receptor–ligand pairs mediated intercellular communication that induce an inflammatory response with different degrees in three BC subtypes, leading to different prognosis. Finally, we constructed subtype-specific prognostic models using cancer cell-specific upregulated genes, and the prognostic models had high predictive performance. In the context of the emphasis on personalized treatment of BC, these prognostic factors, in addition to predicting patient survival, may also serve as some drug targets for clinical treatment, and they provide some ideas for drug design in BC.

## Data Availability

All the data supporting this article are available in the Section 2 and Supplementary Files of the manuscript, or can be obtained by contacting the author.

## Supplementary Materials

The following supporting information can be downloaded at: https://www.mdpi.com/article/10.3390/ijms23179936/s1.
