# Supplementary material for: Delving into the Heterogeneity of Different Breast Cancer Subtypes and the Prognostic Models Utilizing scRNA-Seq and Bulk RNA-Seq
Source: Int J Mol Sci. 2022 Sep 1;23(17):9936. doi: 10.3390/ijms23179936 (PMC9456551; doi:10.3390/ijms23179936)
Supplement: Supplementary file 1 [file ijms-23-09936-s001.zip › ijms-1849646-supplementary.pdf]

Supplementary Table S1. Patient cohort details.

Clinical and pathology details for breast cancer patients analyzed by scRNA-Seq in this study.

Case ID	Gender	Age	Cell Number	Grade	Cancer Type	ER	PR	HER2 IHC	HER2 ISH (ratio)	Subtype
3586	Female	43	6178	3	IDC	100% 2-3+	100% 2-3+	3+	Amplified (6.8)	HER2+ BC
3838	Female	49	2353	3	IDC	0	0	3+	Amplified (8.91)	HER2+ BC
3921	Female	60	3024	3	IDC	0	0	3+	Amplified (10.46)	HER2+ BC
4066	Female	41	5309	2	IDC	70% 3+	0	3+	Amplified (7.7)	HER2+ BC
4517-1	Female	58	2447	3	IDC	0	0	3+	Amplified	HER2+ BC
4040	Female	57	2531	3	IDC	95% 3+	95% 2-3+	0	Non-Amplified	Luminal BC
4398	Female	52	4451	3	IDC	95% 2+	80% 2+	2+	Non-Amplified	Luminal BC
4471	Female	55	8609	2	ILC	100% 3+	100% 3+	0	Non-Amplified	Luminal BC
4530	Female	42	4409	2	IDC	95% 2+	95% 3+	1+	Non-Amplified	Luminal BC
4535	Female	47	3961	2	ILC	95% 3+	70% 2+	2+	Non-Amplified	Luminal BC
3963	Female	61	3527	3	IDC	30% 1+	0	0	Non-Amplified	TNBC
4495	Female	63	7985	3	IDC	0	0	0	Non-Amplified	TNBC
4497-1	Female	49	7986	3	IDC	0	0	0	Non-Amplified	TNBC
4499-1	Female	47	7023	3	IDC	0	0	0	Non-Amplified	TNBC
4515	Female	67	4149	3	IDC	0	0	0	Non-Amplified	TNBC

IDC：Invasive ductal carcinoma ILC：Invasive lobular carcinoma

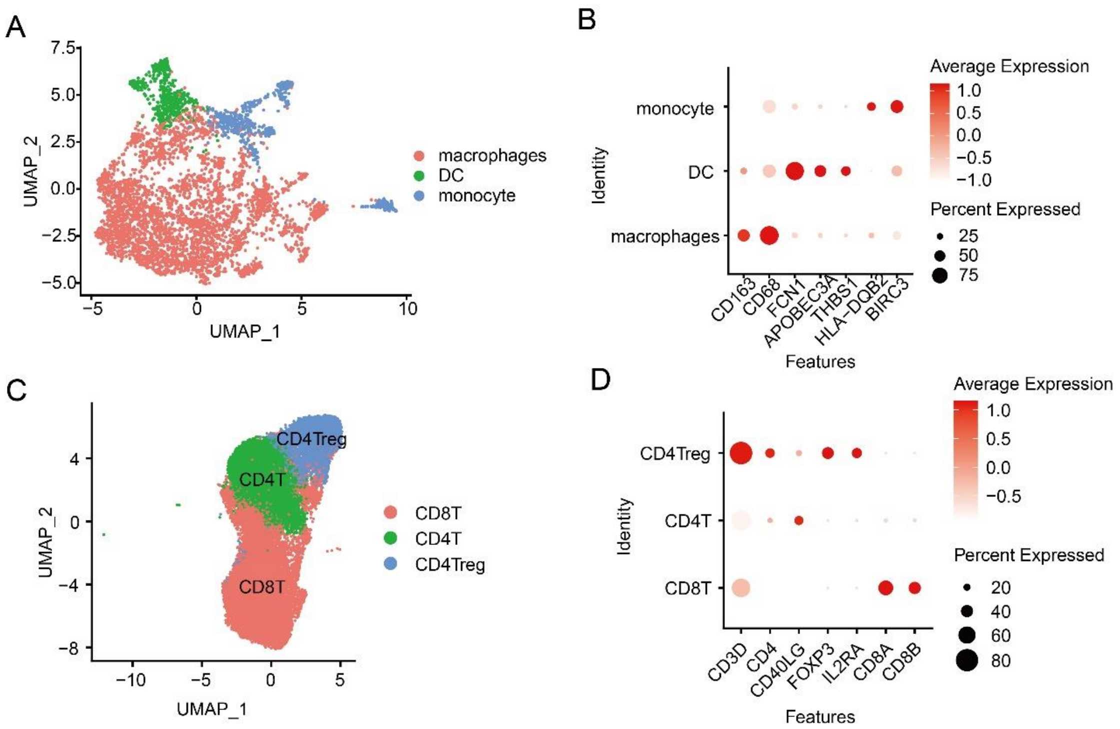

Figure S1. (A) UMAP cluster plot of different myeloid cell types. (B) Expression levels of cellular markers corresponding to different myeloid cell types. (C) UMAP cluster plot of cells colored by different T cell types. (D) Expression levels of cellular markers corresponding to different T cell types.

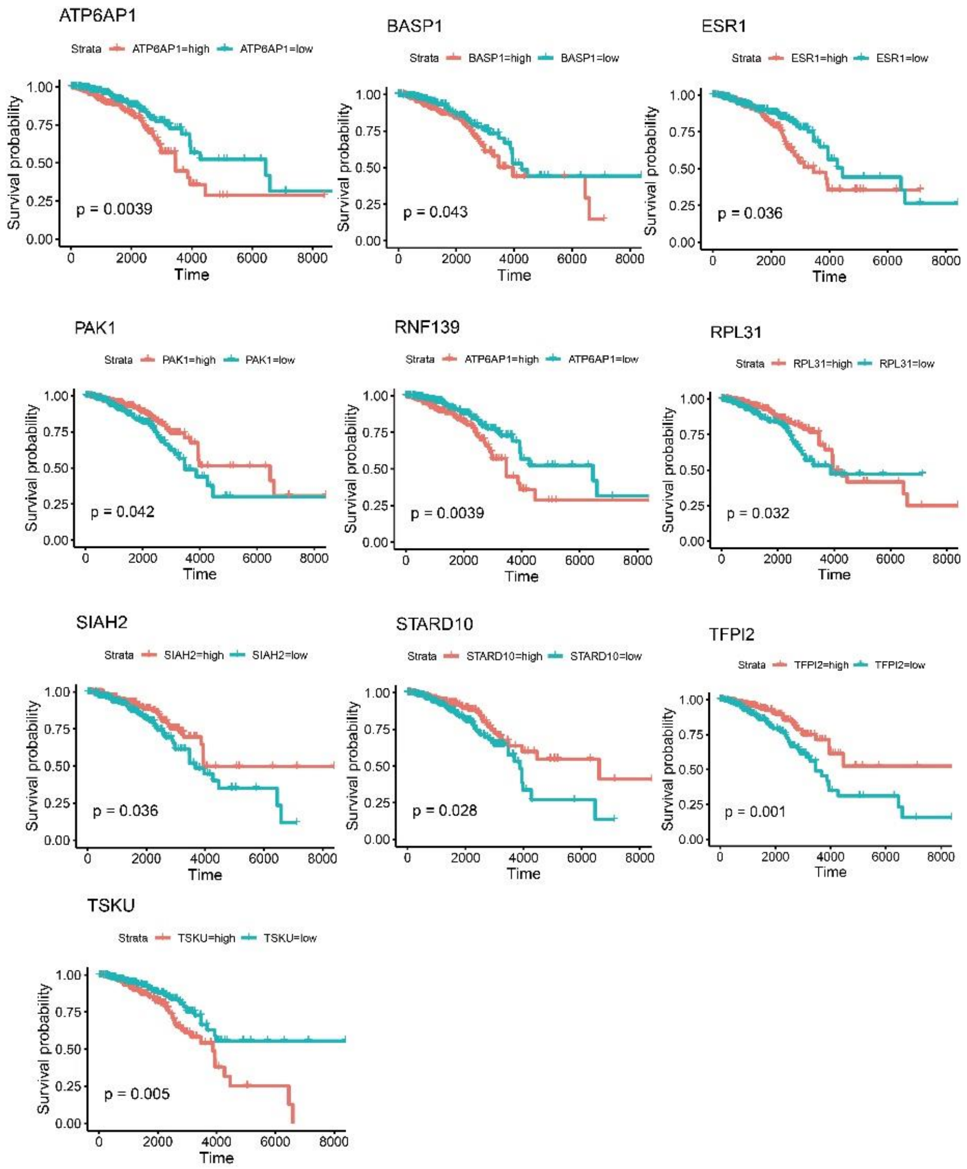

Figure S2. Survival curves of 10 prognostic factors for luminal BC.

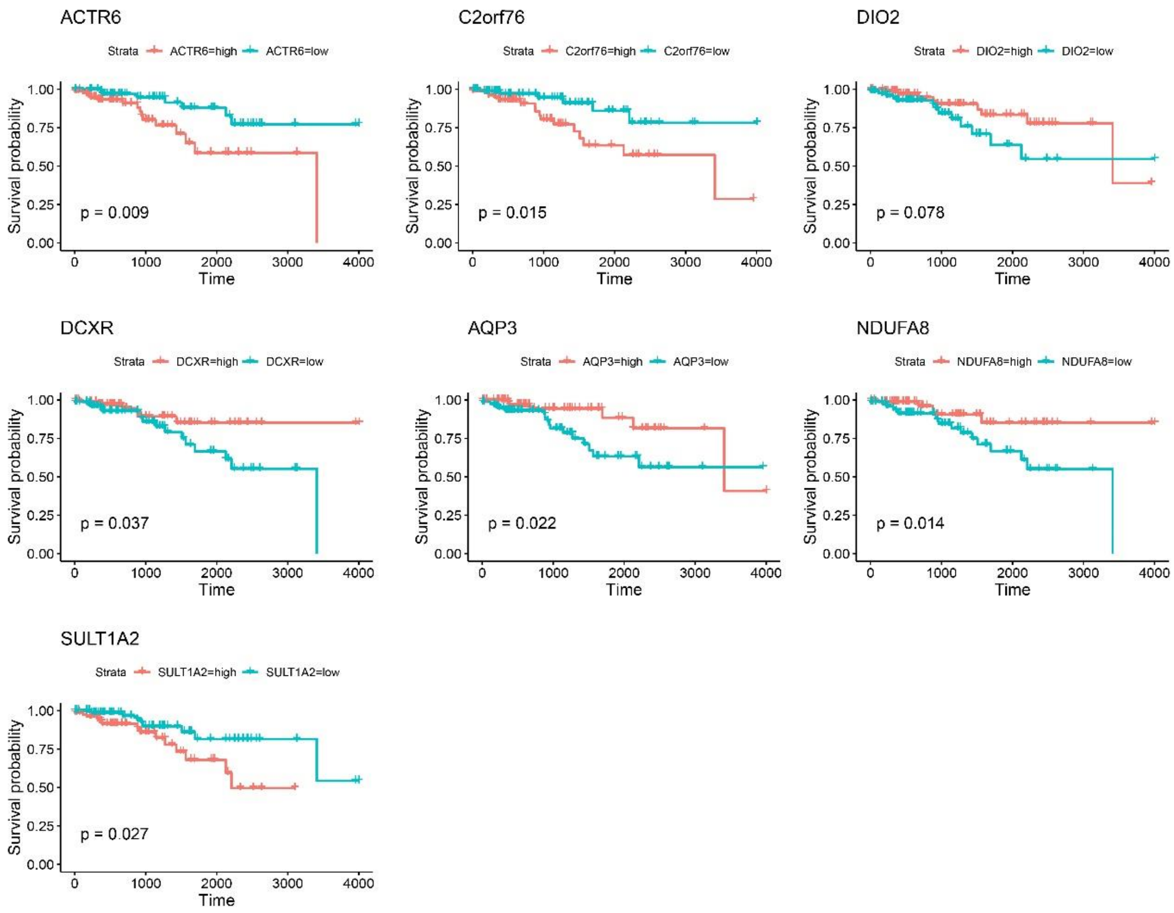

Figure S3. Survival curves of 7 prognostic factors for HER2+ BC.

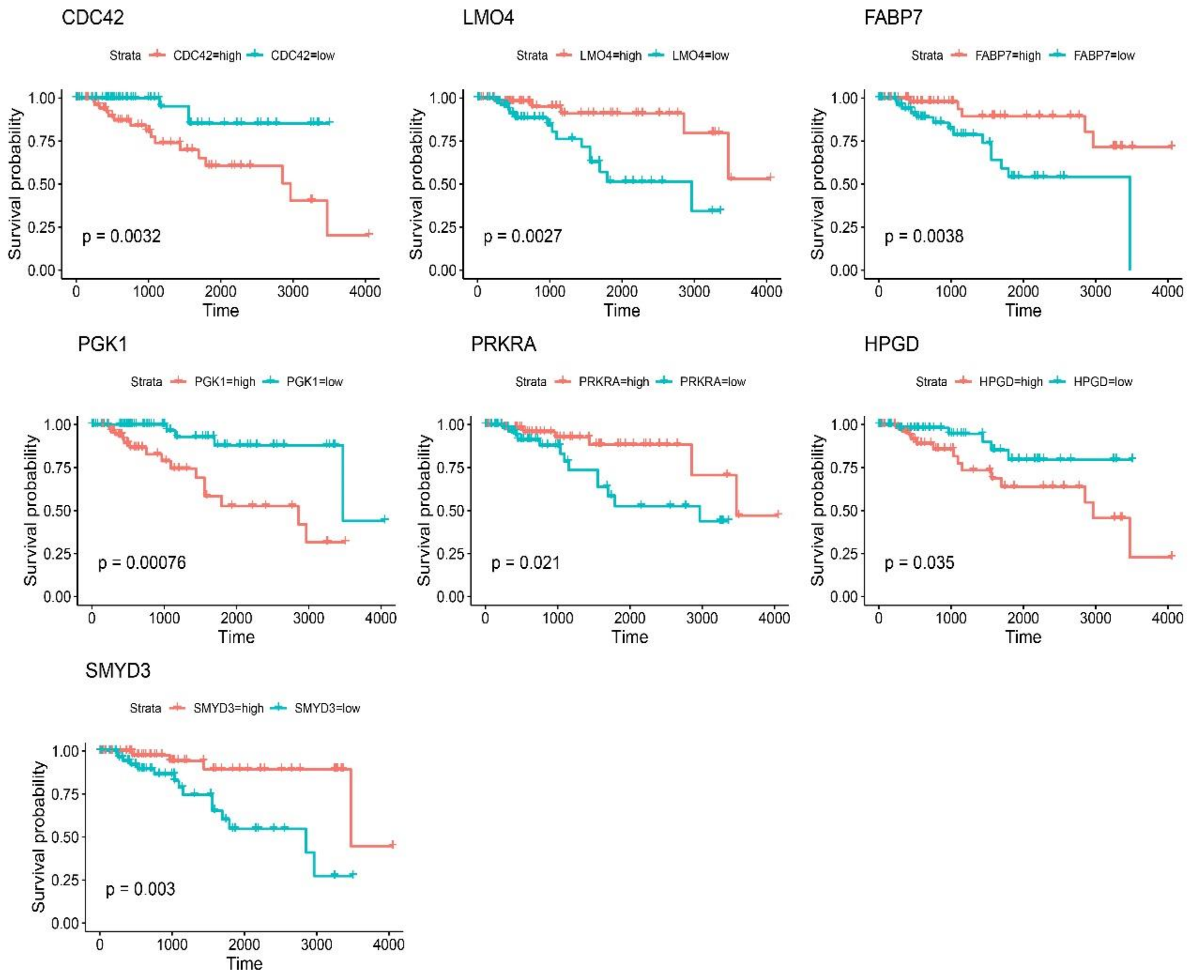

Figure S4. Survival curves of 7 prognostic factors for TNBC.

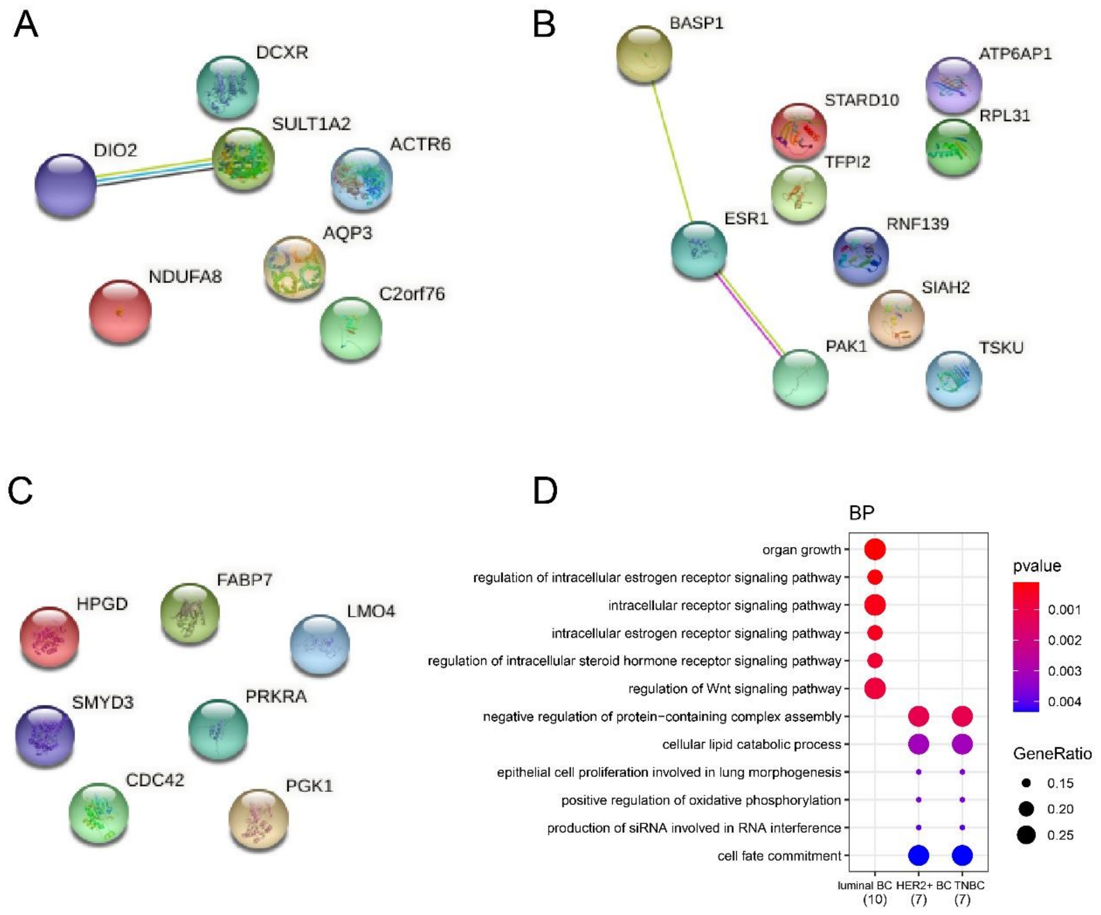

Figure S5. (A-C) The protein-protein interaction networks of prognostic genes of (A) luminal BC subtype; (B) HER2+ BC subtype; (C) TNBC subtype. (D) Functional enrichment analysis of GO biological process by prognostic genes of three subtypes.

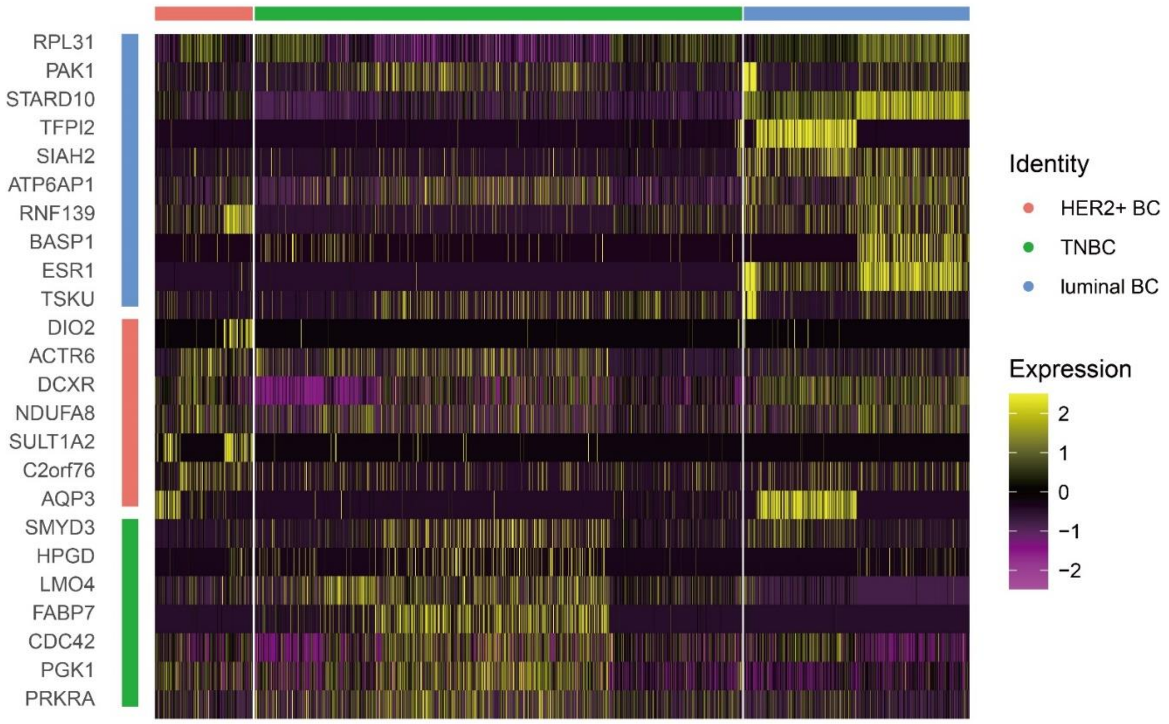

Figure S6. Gene expression profiles of prognostic factors of three BC subtypes in tumor cells of scRNA-seq.
